# Efficacy of acupuncture for patients with Parkinson’s disease–related constipation: study protocol for a randomized controlled, double-blind trial

**DOI:** 10.3389/fnins.2026.1840324

**Published:** 2026-07-22

**Authors:** Shumin Lin, Junwei Lin, Yuhan Li, Xin Liu, Danxia Gu, Fan Zhang, Yixuan Wu, Yuting Wang, Jingqi Fan, Lixing Zhuang

**Affiliations:** 1 The First Clinical School of Medicine, Guangzhou University of Chinese Medicine, Guangzhou, Guangdong, China; 2 Faculty of Geology, Lomonosov Moscow State University, Moscow, Russia; 3 The First Affiliated Hospital of Guangzhou University of Chinese Medicine, Guangzhou, Guangdong, China; 4 Shenzhen Hospital (Fu Tian) of Guangzhou University of Chinese Medicine, Shenzhen, Guangdong, China

**Keywords:** acupuncture, constipation, Parkinson’s disease, randomized controlled trial, sham acupuncture, short chain fatty acid

## Abstract

**Background:**

Parkinson’s disease (PD)-related constipation represents a prevalent and disabling non-motor symptom that imposes a substantial and growing health and economic burden worldwide. Current management strategies, including dietary modification, probiotics, and pharmacological therapies, often provide limited benefit, with potential adverse effects and minimal improvement in other PD-related symptoms. Acupuncture has emerged as a promising complementary therapeutic approach and has demonstrated preliminary clinical efficacy. However, the underlying biological mechanisms and the potential contribution of placebo responses to therapeutic effects remain insufficiently understood.

**Methods:**

This randomized controlled clinical trial will enroll 66 patients with PD–related constipation and randomly assign them in a 1:1 ratio to the real acupuncture group or the sham acupuncture group. Participants, acupuncturists, outcome assessors, and statisticians will be blinded to group allocation. The 8-week trial consists of a 4-week treatment period followed by a 4-week follow-up period. The primary outcome is the longitudinal change in the Chronic Constipation Severity Score (CCS) over time. Secondary clinical outcomes include weekly complete spontaneous bowel movements (CSBMs), spontaneous bowel movements (SBMs), Bristol Stool Scale (BSS) scores, and Unified Parkinson’s Disease Rating Scale (UPDRS) scores. In addition, exploratory outcomes include levels of short-chain fatty acids (SCFAs), histone deacetylase 1 (HDAC1), and phosphorylated *α*-synuclein at serine 129 (pS129-α-syn) measured in biofluid samples. Adverse events will be systematically monitored throughout the trial. The study is conducted in accordance with the SPIRIT guidelines.

**Discussion:**

This trial is designed to evaluate the clinical efficacy of acupuncture for PD-related constipation using a rigorously implemented double-blind, sham acupuncture-controlled design. Although the sham acupuncture procedure may itself induce nonspecific physiological and contextual effects, the use of a standardized double-blind control is intended to reduce expectancy-related bias and improve methodological rigor in the assessment of acupuncture-related outcomes. In addition to clinical symptom evaluation, the integration of SCFAs, HDAC1, and pS129-*α*-syn allows exploratory investigation of potential biological changes associated with treatment. This trial aims to provide multidimensional evidence regarding possible gut–brain axis–related mechanisms involved in acupuncture treatment for PD-related constipation.

**Trial registration:**

The study protocol was registered with the International Traditional Medicine Clinical Trial Registry (ITMCTR 2026000107) on December 7, 2025. The publicly accessible website is: https://itmctr.ccebtcm.org.cn/mgt/search.

## 1 Introduction

Parkinson’s disease (PD) is a progressive neurodegenerative disorder that imposes an increasing burden on healthcare systems worldwide. According to the Global Burden of Disease trial, PD is estimated to affect approximately 11.67 million individuals globally and was responsible for around 420,000 deaths in 2023 (Guo et al., 2025). Constipation is a hallmark non-motor symptom of PD and may affect as many as 70% of patients with PD (Chiang and Lin, 2019). PD-related constipation should not be considered merely a gastrointestinal symptom but rather a clinical feature reflecting overall disease severity. Evidence suggests that patients with PD who experience constipation tend to present with more severe motor impairment and a greater burden of non-motor symptoms and generally exhibit poorer clinical outcomes (Yao et al., 2023; Adams-Carr et al., 2016). In addition, PD-related constipation has been associated with an increased risk of freezing of gait and falls (Mota Telles et al., 2025). Consequently, constipation in PD has emerged as a major public health concern worldwide. Current management strategies for PD-related constipation primarily include dietary modification, probiotics, physical activity, and pharmacological interventions (Ma et al., 2021; Andreozzi et al., 2024; Tan et al., 2021). Although these approaches have demonstrated varying degrees of efficacy in alleviating constipation symptoms, their benefits may diminish with disease progression or prolonged use, and some are associated with adverse effects (Zhang et al., 2023; Yin and Zhu, 2022). At present, there remains a need for additional safe and effective therapeutic options specifically targeting patients with PD-related constipation.

Acupuncture has been used in the management of constipation for several decades, prompting extensive investigation into its clinical efficacy and safety (Li et al., 2023; Wang et al., 2020). A high-quality study published in 2023 demonstrated that, compared with pharmacological therapy, acupuncture significantly improved constipation severity and stool characteristics, and alleviated motor symptoms in patients with PD (Li et al., 2023). Despite the growing body of evidence supporting the beneficial effects of acupuncture for constipation, only a limited number of studies have incorporated sham acupuncture as a control to examine potential placebo effects associated with acupuncture treatment. Blinding of acupuncturists is inherently challenging, and double-blind designs using validated sham acupuncture devices remain limited in studies of PD-related constipation. The underlying biological mechanisms remain incompletely understood. Emerging research has shown that patients with PD–related constipation exhibit alterations in gut microbiota composition accompanied by reduced levels of short-chain fatty acids (SCFAs) (Zhang et al., 2023). However, whether acupuncture exerts therapeutic effects on PD-related constipation through modulation of gut metabolic pathways has not been adequately explored in high-quality clinical studies. Therefore, this carefully designed randomized controlled trial incorporates a double-blind, sham acupuncture-controlled design to investigate the effects of acupuncture on patients with PD-related constipation. In addition, repeated assessments during the 4-week intervention and the subsequent 4-week follow-up will enable a systematic evaluation of the sustained effects of acupuncture on constipation severity and gut-related biomarkers.

## 2 Methods

### 2.1 Trial design and setting

This prospective randomized controlled trial was conducted in accordance with the principles of the Declaration of Helsinki and was developed in compliance with the Standard Protocol Items: Recommendations for Interventional Trials (SPIRIT) 2025 checklist (Chan et al., 2025). The trial is scheduled to be conducted from February 2026 to May 2027 at the outpatient acupuncture clinic of the First Affiliated Hospital of Guangzhou University of Chinese Medicine. The trial protocol has been approved by the hospital’s ethics committee (approval number: JY2025-164) and has been registered with the International Traditional Medicine Clinical Trial Registry (registration number: ITMCTR2026000107).

**Figure 1:**
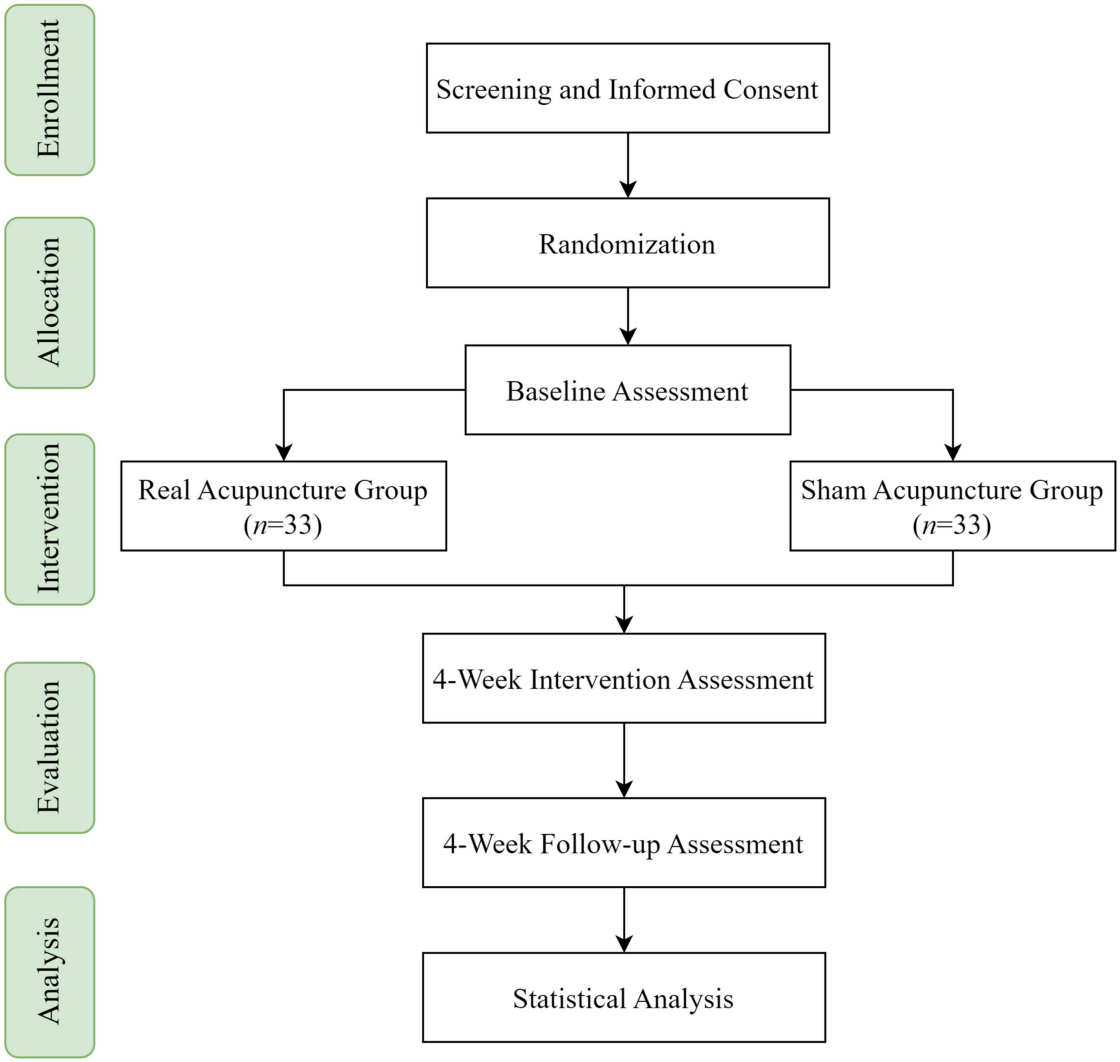
Flow chart of the trial procedure.

A total of 66 eligible participants will be recruited from the acupuncture outpatient department. After providing written informed consent, participants will be randomly assigned in a 1:1 ratio to one of two parallel groups: the real acupuncture group (*n* = 33) or the sham acupuncture group (*n* = 33). The intervention period will last for 8 weeks. Outcome assessments will be conducted at baseline, after the 4-week intervention, and after the 4-week follow-up. A flowchart illustrating the trial design is presented in Figure 1.

### 2.2 Participants

#### 2.2.1 Inclusion criteria

Participants meeting all of the following criteria were eligible for inclusion:

1. Aged 45–80 years, with no restriction on sex.
2. A clinical diagnosis of idiopathic PD according to the Chinese Diagnostic Criteria for Parkinson’s Disease (2016) (Liu, 2016), which requires the presence of bradykinesia combined with either rest tremor or rigidity, along with the absence of absolute exclusion criteria; And a diagnosis of functional constipation according to the Rome IV criteria (Mearin et al., 2016). Specifically, the Rome IV criteria require that patients present with at least two of the following symptoms: straining during more than 25% of defecations; lumpy or hard stools in more than 25% of defecations; sensation of incomplete evacuation; sensation of anorectal obstruction or blockage; manual maneuvers to facilitate defecation; and fewer than three spontaneous bowel movements (SBMs) per week.
3. Hoehn and Yahr (H&Y) stage 1–3 assessed during the “ON” medication state, indicating that patients were in the early-to-middle stages of Parkinson’s disease without severe impairment of balance or walking ability.
4. Continuous treatment with antiparkinsonian medications for at least 6 months, with a stable dosage maintained for a minimum of 2 months before enrollment.
5. No use of medications that may affect gastrointestinal function, including probiotics, within 1 month before treatment. This 4-week washout period is consistent with the transient gut colonization of most probiotics (Zmora et al., 2018) and has been adopted in prior constipation trials (Janssen Duijghuijsen et al., 2024; Yoon et al., 2018).
6. Ability to understand the trial procedures and provision of written informed consent voluntarily.

#### 2.2.2 Exclusion criteria

Participants meeting any of the following criteria were excluded:

1. Presence of severe cognitive impairment or hallucinations that would interfere with reliable completion of clinical assessments.
2. Participation in any other clinical trials involving pharmacological or acupuncture interventions within 1 month before enrollment.
3. Presence of severe comorbid systemic diseases, including but not limited to organic gastrointestinal disorders (e.g., intestinal adhesions, intestinal obstruction, gastrointestinal tumors, or congenital malformations), immune system diseases, cardiovascular or cerebrovascular diseases, or hepatic or renal insufficiency.
4. Previous receipt or planned treatment with deep-brain stimulation (DBS) during the trial period.
5. Pregnant or planning pregnancy, or currently breastfeeding at the time of enrollment.
6. A history of substance abuse (e.g., alcohol or drugs) within the preceding 6 months.
7. A documented history of psychiatric disorders or the presence of psychotic symptoms.
8. Fear of acupuncture that would preclude participation in the intervention.

### 2.3 Sample size calculation

The primary outcome of this trial is the Chronic Constipation Severity Scale (CCS) score. Sample size estimation was based on data from a previous randomized controlled trial investigating acupuncture for patients with PD–related constipation (Lei et al., 2020; Zhen et al., 2021). In that trial, the mean CCS score after acupuncture treatment was 8.79 with a standard deviation (SD) of 4.63, whereas the control group showed a mean CCS score of 12.36 with an SD of 4.26. Sample size calculation was performed using PASS 2021 software, using a superiority test to compare two independent means. The significance level was set at *α* = 0.05 (two-sided), and the statistical power was 80% (1 − *β* = 0.80). Based on these assumptions and an anticipated 20% dropout rate, a total sample size of 66 participants was required to detect a statistically significant superiority in CCS scores between the two groups after the intervention. The sample size was powered for the primary clinical endpoint only.

### 2.4 Randomization and blinding

All participants were randomly assigned in a 1:1 ratio to either the real acupuncture group (*n* = 33) or the sham acupuncture group (*n* = 33). Block randomization was applied, with a block size of four. The random allocation sequence was generated using SPSS Statistics (version 26.0) by an independent statistician who was not involved in clinical implementation or outcome assessment. Allocation concealment was ensured using sequentially numbered, opaque, sealed envelopes. After participants met the eligibility criteria and provided written informed consent, an independent researcher opened the envelopes sequentially to assign participants to the corresponding groups. This trial is designed as a double-blind, randomized controlled trial, with participants, acupuncturists, outcome assessors, and statisticians all blinded to treatment allocation. All procedures will be performed in accordance with predefined standard operating procedures (SOPs).

**Figure 2:**
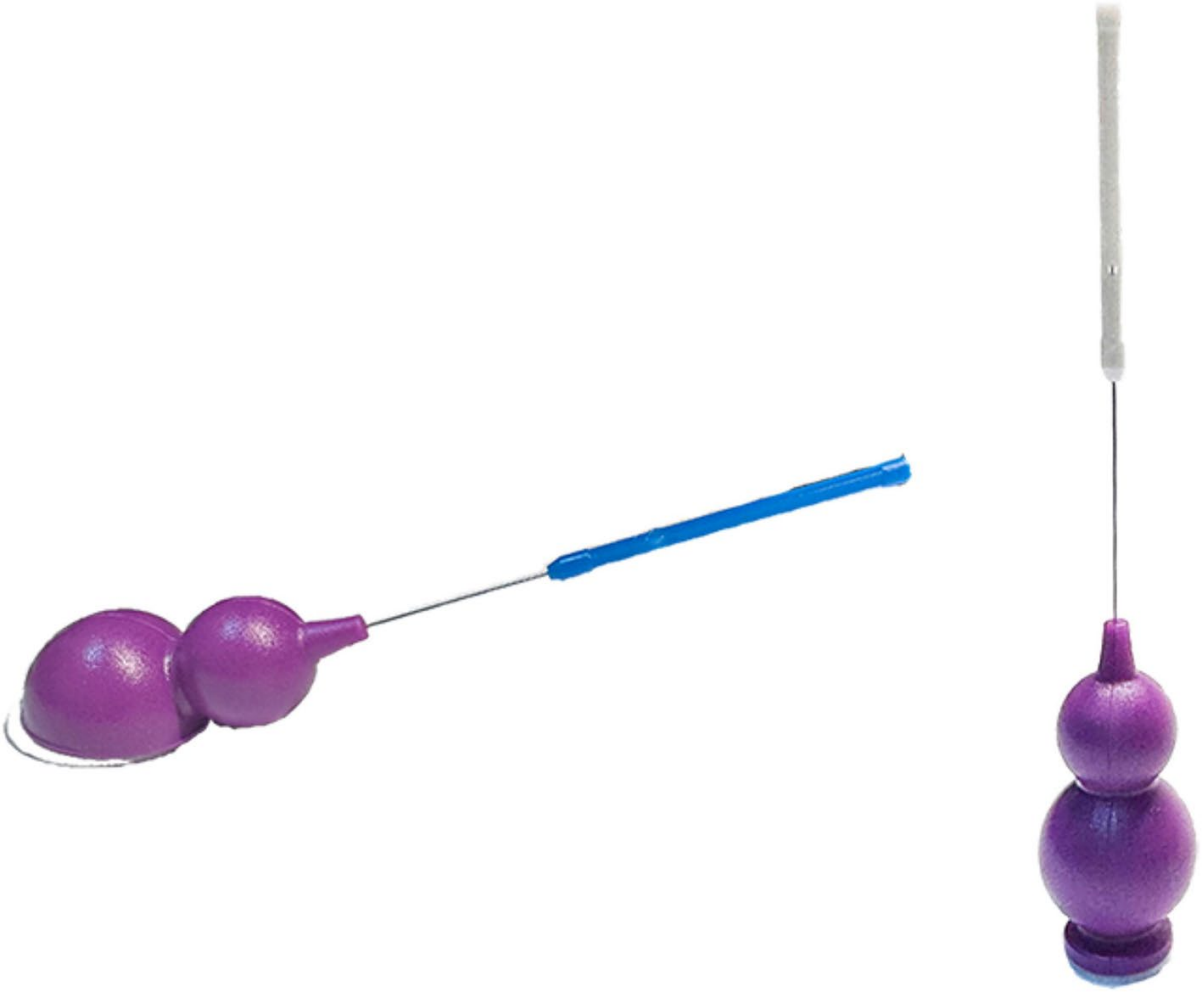
The disposable double-blind acupuncture device.

The acupuncture devices used in this trial were specifically designed and developed by the Zhuang Lixing PD Research Team (Figure 2). The disposable double-blind acupuncture device comprises a fixed blind-spot component and a needle body, and is available in four configurations based on skin penetration and insertion angle: real or sham needles with either vertical or oblique insertion. The real and sham needles are identical in external appearance, featuring an opaque design to prevent visual differentiation. The fixed blind-spot component is gourd-shaped and equipped with a custom adhesive base, allowing stable attachment to the skin surface. For real acupuncture, the needle tip is sharp, and a small slit at the bottom of the fixed blind-spot component allows the needle body (diameter × length: 0.35 × 50 mm or 0.35 × 65 mm) to penetrate the skin and elicit the intended acupuncture effect. In contrast, the sham needles have blunt tips and shorter needle bodies (0.35 × 42 mm or 0.35 × 36 mm), preventing skin penetration. However, the sham needles are sufficiently long to produce a mild pain, thereby maintaining participant blinding. In addition, a friction mechanism within the fixed blind-spot component provides resistance during insertion, preventing acupuncturists from distinguishing real from sham needles and thereby ensuring operator blinding. To evaluate the preliminary feasibility of operator blinding, a pilot validation test was conducted prior to the main trial. Ten acupuncturists with over 2 years of clinical experience performed the procedures and were asked to guess the needle type (real, sham, or uncertain). The distribution of responses did not suggest a clear ability to reliably distinguish between real and sham needles. Although limited by the small sample size, these findings provided preliminary support for the feasibility of operator blinding.

The acupuncture device used in this trial has been granted a Chinese utility model patent (Patent No. 202520263962. X) and will be manufactured by a qualified company (laozongyi Co., Ltd.). Outcome assessors and statisticians will remain unaware of group allocation throughout the trial to minimize assessment and analysis bias. The success of blinding will be assessed after completion of the intervention by asking participants and acupuncturists to guess the allocated treatment group, and the distribution of responses will be compared with chance levels. To formally and quantitatively evaluate blinding success over the course of the trial, Bang’s Blinding Index (BBI) will be utilized. Within 5 min after the first treatment session and at the completion of the final session, both participants and acupuncturists will be asked to guess the group allocation (real, sham, or uncertain). The BBI will then be calculated to statistically assess the success and direction of blinding for both patients and practitioners.

### 2.5 Intervention

#### 2.5.1 Real acupuncture group

**Figure 3:**
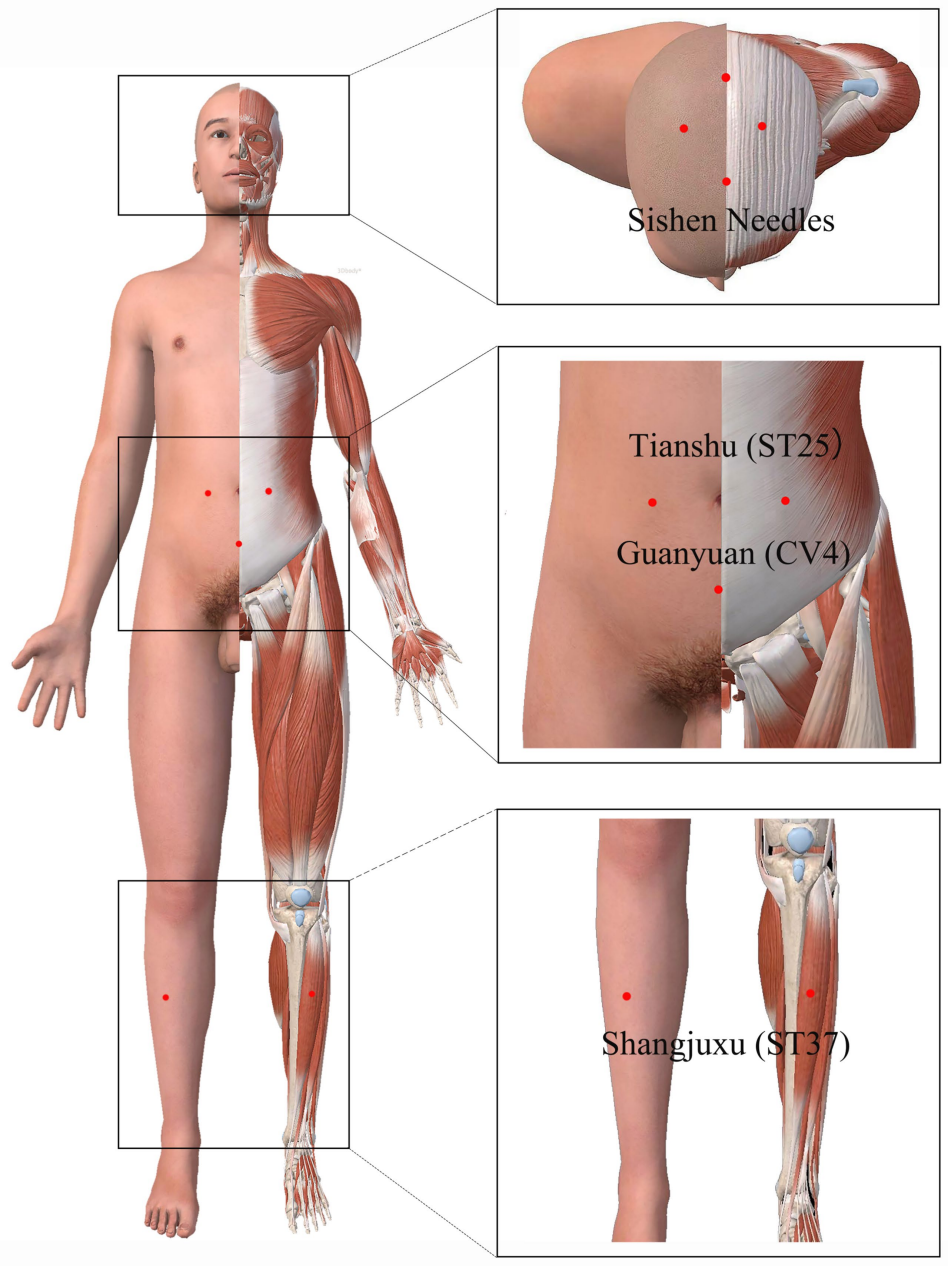
The illustration of acupoints used in this trial.

Participants allocated to the real acupuncture group will receive standard antiparkinsonian pharmacotherapy in accordance with the Chinese Guidelines for the Treatment of Parkinson’s Disease (Fourth Edition) (Chen, 2020). During the treatment period, participants will maintain their baseline antiparkinsonian medication regimen. Any necessary adjustments in medication dosage or regimen will be documented in detail in the case report forms (CRFs). In addition, all participants will be advised to follow a Mediterranean-style diet with a high fiber content (Portugal-Nunes et al., 2021). Dietary adherence will be monitored at each treatment visit through patient interviews, and dietary recommendations will be reinforced as needed to ensure compliance. On this basis, participants will undergo real acupuncture treatment at a frequency of three sessions per week, with each session lasting 30 min, for four consecutive weeks. The acupuncture prescription consists of Sishen Needles and the Intestinal Three-Needle (Tianshu [ST25], Guanyuan [CV4], and Shangjuxu [ST37]) (Table 1; Figure 3). The anatomical locations of all acupoints will strictly follow the National Standard of the People’s Republic of China (GB/T 1236–2021) (Xiaodong and Longxiang, 2022). All acupuncture procedures will be performed by licensed acupuncturists who have received standardized training and have at least 2 years of clinical experience in acupuncture practice.

**Table 1:** Location of acupoints used in this trial.

Acupoints	Location
Sishen needles	Four acupoints are located 1.5 B-cun anterior, posterior, and lateral to Baihui (GV20).
Tianshu (ST25)	On the upper abdomen, 2 B-cun lateral to the centre of the umbilicus.
Guanyuan (CV4)	On the lower abdomen, 3 B-cun inferior to the centre of the umbilicus, on the anterior median line.
Shangjuxu (ST37)	On the anterior aspect of the leg, on the line connecting ST35 with ST41, 6 B-cun inferior to ST35.

#### 2.5.2 Sham acupuncture group

Participants in the sham acupuncture group will receive the same standard antiparkinsonian pharmacotherapy and identical dietary counseling as those in the real acupuncture group. The only difference between the two groups lies in the acupuncture intervention. In the sham acupuncture group, needles will be applied without skin penetration. The selected acupoints, treatment frequency, session duration, and total number of sessions will be identical to those used in the real acupuncture group. Sham acupuncture procedures will follow the same procedural protocols to ensure consistency across groups.

### 2.6 Emergency treatment

Participants will be permitted to use rescue emergency treatment if they experienced no complete spontaneous bowel movements for more than three consecutive days, or developed intolerable symptoms related to constipation, such as abdominal distension. Rescue medications included oral lactulose solution or glycerin enema. Each use of rescue treatment was fully documented in the CRFs. Bowel movements occurring within 24 h after rescue treatment will not be included in the calculation of weekly complete spontaneous bowel movements (CSBMs) or weekly spontaneous bowel movements (SBMs).

### 2.7 Outcomes assessment

Outcome evaluation primarily focused on two domains: constipation severity and other PD-related outcomes. Clinical scale assessments and the collection of fecal and blood samples were conducted at three time points: pre-treatment (baseline), after week 4 of the treatment period, and at week 4 of the follow-up period (Table 2). All assessments and biospecimen collections were performed during the participants “on” medication state at each visit. Outcome assessors received standardized training and remained blinded to group allocation throughout the trial.

**Table 2:** Details of the planned visit schedule.

Task	Baseline period	Treatment period				Follow-up period
	Week 0	Week 1	Week 2	Week 3	Week 4	Week 8
Enrollment						
Informed consent	√					
Random assignment	√					
Demographic characteristics	√					
Intervention						
Real acupuncture		√	√	√	√	
Sham acupuncture		√	√	√	√	
Assessments						
CCS	√				√	√
CSBMs and SBMs	√				√	√
BSS	√				√	√
UPDRS	√				√	√
SCFAs	√				√	√
HDAC1 and pS129-α-syn	√				√	√
Blinding assessment		√			√	
Safety						
Adverse events		√	√	√	√	√

#### 2.7.1 Primary outcome

The Chronic Constipation Severity Score (CCS) was used as the primary outcome measure to quantify constipation severity. The CCS evaluates eight dimensions of constipation, including bowel movement frequency, degree of defecation difficulty, sensation of incomplete evacuation, presence of abdominal pain or bloating, maximum time required for each defecation episode, use of defecation assistance, frequency of unsuccessful defecation urges within 24 h, and duration of constipation. Total scores range from 0 to 30, with higher scores indicating more severe constipation symptoms (Higashimori et al., 2021). To provide a clinically interpretable anchor for treatment response, a reduction of ≥5 points from baseline in the CCS was prespecified as a threshold for clinically meaningful improvement based on previous studies in neurogenic and refractory constipation populations (Kumar et al., 2017; Preziosi et al., 2011). However, we acknowledge that a universally established MCID for PD-related constipation is currently unavailable.

#### 2.7.2 Secondary outcomes

##### 2.7.2.1 Weekly CSBMs and SBMs

CSBMs were defined as bowel movements occurring without laxatives or manual assistance and accompanied by a sensation of complete evacuation. SBMs were defined as bowel movements occurring without laxatives or manual assistance, regardless of completeness. Lower weekly frequencies indicate greater constipation severity.

##### 2.7.2.2 Bristol Stool Scale (BSS) score

Stool consistency was evaluated using the Bristol Stool Scale (BSS), which classifies stool form into seven types, ranging from separate hard lumps (Type 1) to watery stools (Type 7) (Lai et al., 2023).

##### 2.7.2.3 Unified Parkinson’s Disease Rating Scale (UPDRS)

Parkinson’s disease severity was assessed using the Unified Parkinson’s Disease Rating Scale (UPDRS), a widely used clinical instrument that consists of four parts (I–IV) assessing mentation, activities of daily living, motor examination, and complications. Higher total scores reflect greater disease severity.

#### 2.7.3 Exploratory outcomes

As the study was not formally powered for mechanistic biomarker endpoints, all analyses involving these biomarkers were predefined as strictly exploratory and hypothesis-generating.

##### 2.7.3.1 Short-chain fatty acids (SCFAs)

Fecal samples were collected and stored at −80 °C within 5 h of collection. Concentrations of SCFAs were quantitatively analyzed using an ultra-performance liquid chromatography–tandem mass spectrometry (UPLC–MS/MS) system.

##### 2.7.3.2 Histone deacetylase 1 (HDAC1) and phosphorylated *α*-synuclein at serine 129 (pS129-*α*-syn)

Fecal and blood samples were collected and processed immediately after collection. Blood samples were centrifuged at 3,000 ×*g* for 15 min at 4 °C to obtain cell-free serum. Fecal samples were homogenized in phosphate-buffered saline (PBS, pH 7.4) and centrifuged at 12,000 ×*g* for 20 min at 4 °C to obtain clarified supernatants free of cellular debris. The resulting supernatants were aliquoted and stored at −80 °C until analysis.

The concentrations of histone deacetylase 1 (HDAC1) and phosphorylated *α*-synuclein at serine 129 (pS129-α-syn) were measured using commercially available enzyme-linked immunosorbent assay (ELISA) kits according to the manufacturers’ instructions. The HDAC1 assay was performed using a human HDAC1 ELISA kit (Cat. No. JL20104-96 T; Jianglai Biotechnology, Shanghai, China), and the pS129-α-syn assay was performed using a human phosphorylated α-synuclein (Ser129) ELISA kit (Cat. No. H712234-96 T/EA; Macklin Biochemical, Shanghai, China).

Optical density was measured at 450 nm using a multimode microplate reader, and protein concentrations were calculated according to standard curves following the manufacturers’ instructions.

#### 2.7.4 Blinding assessment and safety monitoring

To assess the success of blinding, participants and acupuncturists will independently indicate whether they believe the intervention was real or sham acupuncture after the first treatment session and at the completion of the final session. Their responses will be recorded using a standardized blinding assessment form and analyzed using BBI and descriptive statistics to assess the effectiveness of the blinding procedure.

Safety monitoring will focus on adverse events (AEs) related to acupuncture. All AEs will be recorded in standardized case report forms, including time of onset, duration, severity, suspected relationship to the intervention, actions taken, and clinical outcomes. AEs will be graded and their causality assessed in accordance with the Record Specification on Adverse Events in Clinical Trials of Acupuncture and Moxibustion (T/CAAM 0002-2022), ensuring a standardized and objective evaluation framework for acupuncture interventions (Moxibustion CAoAa, 2022). All AEs will be reviewed in a timely manner and reported to the Ethics Committee of the First Affiliated Hospital of Guangzhou University of Chinese Medicine in line with institutional and regulatory requirements. Serious adverse events (SAEs) will be reported immediately and managed according to predefined safety management protocols.

## 3 Quality control, data management, and monitoring

All data will be recorded by investigators or outcome assessors using CRFs, while patient-reported outcomes (e.g., Weekly CSBMs, SBMs and BSS score) will be collected using paper-based diaries. To ensure data accuracy and completeness, all CRFs and paper diaries will undergo independent secondary verification by designated quality control personnel. An independent monitoring committee from the coordinating institution will conduct regular audits throughout the trial. These audits will include source data verification of CRFs and random contact with a subset of participants to confirm the authenticity and consistency of recorded information. All source documents, including patient diaries and CRFs, will be maintained in a complete, timely, legible, and traceable manner in accordance with Good Clinical Practice (GCP) guidelines. Any corrections to the recorded data will not obscure the original entry; instead, amendments will be documented with a written explanation, dated, and signed by the responsible investigator. Only CRFs and verified diaries reviewed and approved by the clinical monitor will be eligible for data entry. A designated data manager will oversee data entry and management. To ensure data integrity, double data entry will be independently performed by two trained personnel, followed by cross-validation and resolution of discrepancies before database lock. All trial data will be securely stored with restricted access, and the database will be locked only after completion of all data queries and quality checks.

## 4 Statistical analysis

All analyses were conducted according to the intention-to-treat (ITT) principle. The full analysis set (FAS) included all participants who were randomized and received at least one session of treatment. Statistical analyses were performed using SPSS Statistics software (version 26.0). Continuous variables were tested for normality using the Shapiro–Wilk test. Normally distributed data are presented as mean ± standard deviation (SD), whereas non-normally distributed data are expressed as median (interquartile range, IQR). Between-group comparisons were performed using the independent-samples t-test or Mann–Whitney U test, as appropriate. Within-group comparisons were conducted using the paired t-test or Wilcoxon signed-rank test. Categorical variables were analyzed using the Chi-square test. Pearson or Spearman correlation analyses were performed, as appropriate, to evaluate the correlations of SCFAs, HDAC1, and pS129-*α*-syn levels with CCS scores.

Repeated-measures data were analyzed using linear mixed-effects models (LMMs). Fixed effects included group, time, and the group-by-time interaction. Predefined covariates comprised age, sex, disease duration, and levodopa equivalent daily dose (LEDD). Participant-specific random intercepts were included as random effects. The covariance structure for repeated measures was selected based on the Akaike Information Criterion (AIC) and Bayesian Information Criterion (BIC), and the optimal covariance structure was used for model estimation. If the group-by-time interaction was statistically significant, *post hoc* multiple comparisons were performed with Bonferroni correction, and the results were presented as least squares mean differences (LSMDs) with 95% confidence intervals (CIs). Sensitivity analyses, including complete-case analysis and multiple imputation, were conducted to evaluate the impact of missing data on the robustness of the findings. No adjustment for multiple comparisons was applied for exploratory endpoints, including SCFAs, HDAC1, and pS129-*α*-syn; therefore, these findings should be interpreted cautiously pending further validation. All statistical tests were two-sided, and *p* < 0.05 was considered statistically significant.

## 5 Discussion

Constipation is highly prevalent among patients with PD (Knudsen et al., 2017). Accumulating evidence indicates that constipation is closely associated with both the onset and progression of PD. Patients with PD-related constipation tend to exhibit more severe motor impairment and a higher burden of non-motor symptoms. This association suggests that constipation may reflect a more advanced or widespread neurodegenerative process rather than an isolated peripheral symptom (Camacho et al., 2021). Importantly, PD-related constipation differs fundamentally from functional constipation observed in the general population. Emerging evidence suggests that PD-related constipation is partly attributable to enteric nervous system dysfunction and dopaminergic signaling alterations, resulting in impaired gastrointestinal motility that closely parallels motor dysfunction (Singaram et al., 1995). As motor symptoms worsen, reduced physical activity and increased use of antiparkinsonian medications may further aggravate constipation. In turn, impaired gastrointestinal motility can reduce intestinal drug absorption, potentially diminishing therapeutic efficacy and creating a vicious cycle.

Current therapeutic strategies for PD-related constipation primarily focus on promoting gastrointestinal motility to alleviate bowel symptoms. However, these approaches may be associated with adverse effects and generally provide limited improvement in other PD-related symptoms (Johanson and Kralstein, 2007). Acupuncture, a simple, safe, and widely accepted complementary therapy, has shown preliminary clinical efficacy in the treatment of PD-related constipation (Li et al., 2023). However, current research in this field is characterized by two major limitations. First, most randomized controlled trials have used levodopa therapy alone or levodopa combined with prokinetic agents as control interventions. Only a small proportion of studies have accounted for the potential placebo effects of acupuncture by employing sham acupuncture as a control condition. To our knowledge, no double-blind clinical randomized controlled trials investigating acupuncture for PD-related constipation have been identified in publicly available databases. This absence may partly reflect the procedural characteristics of acupuncture, for which acupuncturist blinding is inherently difficult to implement, thereby introducing a potential risk of bias. Second, most existing studies have relied primarily on subjective clinical rating scales as primary outcome measures, without incorporating objective biological markers or further exploring the underlying mechanisms of therapeutic effects. Exclusive reliance on subjective outcomes is susceptible to placebo effects, expectancy bias, and assessor bias and may obscure potential discrepancies between symptomatic improvement and the underlying pathological processes.

Against this background, the trial is designed as a high-quality randomized controlled trial to minimize the influence of placebo effects and expectancy bias, while simultaneously exploring potential mechanisms involving gut metabolic pathways. Based on prior evidence and clinical expertise, Sishen Needle and Intestinal Three-Needle (Tianshu [ST25], Guanyuan [CV4], and Shangjuxu [ST37]) were selected as the intervention acupoint sets. Both represent classic and widely applied components of Jin’s three-needle system and have demonstrated clinical relevance in the treatment of gastrointestinal dysfunction (Li et al., 2023; Li et al., 2023).

To address methodological limitations in previous acupuncture studies, this trial innovatively employs a disposable double-blind acupuncture device developed by Professor Lixing Zhuang’s research team. Identical intervention procedures are applied in both the real and sham acupuncture groups, with the only difference being the type of needle incorporated within the device. The real and sham devices are indistinguishable in appearance, and an embedded friction mechanism simulates tissue resistance during needle insertion, preventing acupuncturists from identifying needle type based on tactile feedback or visual cues. Furthermore, the sham needles are precisely engineered with controlled length and a smooth blunt tip, allowing participants to perceive mild pricking sensations without actual skin penetration. This design supports a double-blind approach in acupuncture trials and may help reduce subjective bias from both practitioners and outcome assessors while improving the rigor of placebo-controlled evaluation. However, it must be acknowledged that both real and sham acupuncture interventions may induce nonspecific physiological, placebo-related, and contextual effects, which should be considered when interpreting the results. As a consequence, between-group differences may be attenuated, and observed effects may not fully reflect the specific effects of acupuncture. Despite this limitation, the trial is expected to provide a more objective assessment of both the clinical efficacy and the associated biological changes of acupuncture in PD-related constipation. In addition, clinical biofluid samples are incorporated to capture objective biological responses to treatment. Given the central role of the gut in PD pathogenesis, fecal samples are collected to reflect gut microbial function associated with constipation. SCFAs, HDAC1, and pS129-*α*-syn are measured to evaluate changes in microbial metabolism, epigenetic regulation, and local pathological α-syn. Peripheral blood samples are simultaneously analyzed for HDAC1 and pS129-α-syn to explore potential systemic biomarker changes associated with acupuncture intervention.

Since early reports in 2015 linking PD to gut microbiota dysbiosis, accumulating evidence has highlighted microbial alterations and their potential contribution to disease pathophysiology (Scheperjans et al., 2015). The pathogenesis of PD-related constipation is increasingly studied within the framework of the microbiota–gut–brain axis. SCFAs, as the key microbial-derived metabolites, have consequently emerged as potential regulatory mediators in this context (Reichardt et al., 2018). Patients with PD-related constipation exhibit a reduced abundance of SCFAs-producing bacterial taxa, accompanied by decreased fecal SCFAs concentrations, indicating disruption of the intestinal microbial ecosystem (Xiao et al., 2022). SCFAs, particularly butyrate and propionate, regulate intestinal immune homeostasis in part through inhibition of histone deacetylases (HDACs), thereby modulating gene expression (Chiang and Lin, 2019). A reduction in SCFA availability has been proposed to be associated with increased HDAC1 activity, which may influence epigenetic regulation and is thought to promote pro-inflammatory responses. Disease severity in PD has been reported to correlate negatively with fecal SCFAs levels and positively with HDAC1 activity, supporting a potential link between microbial metabolic dysfunction and disease progression (Shin et al., 2020; Eshleman et al., 2024; Diniz et al., 2025). Intestinal inflammation and increased gut permeability may enhance host exposure to microbial antigens and endotoxins, thereby promoting persistent low-grade systemic inflammation (Sampson et al., 2016; Clairembault et al., 2015). Such inflammatory signaling is thought to facilitate pathological post-translational modifications of *α*-syn, particularly pS129-α-syn (Reimer et al., 2018). Studies have shown that up to 90% of α-syn in Lewy bodies in the brains of patients with PD is pS129-*α*-syn. Compared with other α-syn species, pS129-α-syn has been reported to exhibit greater propagation potential in biological environments (Ma et al., 2016). These findings suggest a potential association between gut-derived inflammatory processes and α-syn-related pathology in PD. However, the precise relationship between these pathways and neurodegenerative progression remains incompletely understood. Accordingly, this randomized double-blind controlled trial collects fecal and blood samples to quantify changes in SCFAs, HDAC1, and pS129-α-syn following acupuncture intervention in patients with PD-related constipation. These biomarker analyses are exploratory in nature and are intended to provide preliminary observational evidence regarding potential microbiota–gut–brain axis–related changes associated with acupuncture intervention. Given the limited sample size and exploratory design of this trial, causal relationships between biomarker alterations and clinical outcomes cannot be established. Accordingly, these findings warrant further investigation in larger, well-designed studies to validate these exploratory observations.

Furthermore, several limitations of this trial should be acknowledged. First, this is a single-center study recruiting patients with PD-related constipation primarily from southern China, with a relatively modest sample size. Consequently, the findings may have limited generalizability to advanced PD populations, different ethnic groups, and non-Chinese healthcare settings. Future multicenter studies incorporating more diverse demographic cohorts and disease stages are warranted to improve the external validity of the findings. Second, the 4-week follow-up period may be insufficient to fully characterize the long-term and sustained effects of acupuncture in this patient population. Therefore, future studies should incorporate longer follow-up durations and additional assessment time points to better evaluate the durability of treatment effects. Third, although SCFA levels are assessed to explore gut–brain axis–related metabolic changes, concurrent microbiome profiling is not included in the current analysis framework. We acknowledge that this may limit the depth of mechanistic interpretation of SCFA-related findings. Finally, the potential for experienced acupuncturists to become unblinded over the course of prolonged treatment remains a limitation. Further refinement of sham acupuncture devices may help improve blinding integrity in future trials.

Given the relatively high frequency of trial visits, all participants will receive standardized Mediterranean diet counseling, regular assessments of PD status, and appropriate rescue treatment for constipation when clinically indicated throughout the trial period. Upon completion of the 8-week trial, participants allocated to the sham acupuncture group will be offered up to 12 sessions of real acupuncture treatment voluntarily as post-trial compensation.
